# Isogenic human pluripotent stem cell disease models reveal ABRA deficiency underlies cTnT mutation-induced familial dilated cardiomyopathy

**DOI:** 10.1007/s13238-021-00843-w

**Published:** 2021-04-22

**Authors:** Bin Li, Yongkun Zhan, Qianqian Liang, Chen Xu, Xinyan Zhou, Huanhuan Cai, Yufan Zheng, Yifan Guo, Lei Wang, Wenqing Qiu, Baiping Cui, Chao Lu, Ruizhe Qian, Ping Zhou, Haiyan Chen, Yun Liu, Sifeng Chen, Xiaobo Li, Ning Sun

**Affiliations:** 1 Department of Physiology and Pathophysiology, State Key Laboratory of Medical Neurobiology, School of Basic Medical Sciences, Fudan University, Shanghai, 200032 China; 2 Shanghai Institute of Precision Medicine, Ninth People’s Hospital, Shanghai Jiao Tong University School of Medicine, Shanghai, 200025 China; 3 Shanghai Key Lab of Birth Defect, Children’s Hospital of Fudan University, Shanghai, 201102 China; 4 Shanghai Key Laboratory of Clinical Geriatric Medicine, Research Center on Aging and Medicine, Fudan University, Shanghai, 200032 China; 5 Department of Echocardiography, Zhongshan Hospital, Fudan University, Shanghai, 200032 China; 6 Department of Biochemistry, School of Basic Medical Sciences, Fudan University, Shanghai, 200032 China

**Dear Editor,**

Dilated cardiomyopathy (DCM) is a common form of inherited cardiomyopathy. In the past decades, single mutations in various genes encoding sarcomeric, cytoskeletal, and channel proteins etc. have been found to be associated with DCM (Hershberger et al., 2013; McNally and Mestroni, 2017). However, the mechanisms how single mutations in sarcomeric or structural genes lead to the disease remain elusive. An interesting phenomenon often seen in familial cardiomyopathy is that different single mutations on the same gene can cause either DCM or hypertrophic cardiomyopathy (HCM) (Kathiresan and Srivastava, 2012), which exhibit almost opposite disease phenotypes. DCM is characterized by thinned myocardium and septum, ventricular chamber dilation, and systolic dysfunction (Jefferies and Towbin, 2010; McNally and Mestroni, 2017), while HCM exhibits thickened myocardium and septum, reduced ventricular chamber, and diastolic dysfunction (Richard et al., 2003). At the cellular level, HCM cardiomyocytes exhibit concentric hypertrophy characterized by assembly of myofilaments in parallel and widening of the myocytes. In contrast, DCM cardiomyocytes show eccentric hypertrophy, with assembly of the myofilaments in series and myocyte elongation (Kehat and Molkentin, 2010).

The gene *TNNT2*, which encodes cardiac troponin T (cTnT), is one of such gene showing ramification of cardiomyopathy phenotypes. Deletion of lysine 210 (ΔK210) in one allele of *TNNT2* was found to cause familial DCM. Patients carrying this heterozygous mutation exhibit dilated ventricular chamber and systolic dysfunction leading to progressive heart failure with high mortality (Kamisago et al., 2000). In contrast, deletion of glutamic acid 160 (ΔE160) in one allele of *TNNT2* causes HCM (Watkins et al., 1995). Previous studies showed that the ΔK210 mutation and ΔE160 mutation causes an opposite myofilament Ca^2+^ sensitivity and force generation (Morimoto et al., 2002), possibly leading to opposite macroscopic disease phenotypes. The contrast cardiomyopathy phenotypes arisen from these two different cTnT mutations provide us a nice model to explore detailed molecular basis triggering DCM disease development.

To investigate the key factors involved in this disease divergence, we generated isogenic human embryonic stem cell (hESC) lines carrying the cTnT-ΔK210 or cTnT-ΔE160 mutation by TALEN-mediated genomic engineering (Fig. S1A and S1B). This eliminated the genetic variations of induced pluripotent stem cells (iPSCs) even from individuals from the same family. Southern blotting using a probe targeting the PGK-Puro cassette confirmed that no non-specific integration within the genome (Fig. S1C–E). Sanger’s sequencing analyses of each of these cell lines further confirmed their heterozygous and homozygous nature (Fig. 1A). We next differentiated the heterozygous and homozygous cTnT-∆K210 and -∆E160 hESCs toward the cardiac lineage. cTnT-∆K210 and -∆E160 hESC-cardiomyocytes showed abnormal beating activities (Fig. 1B), which suggested a correlation with cardiac arrhythmia. To examine whether ∆K210 and ∆E160 mutations have an impact on myofilament organization, we further analyzed cardiomyocytes derived from these mutant hESCs by immunostaining the sarcomeric proteins cTnT and α-actinin. Compared to WT hESC-cardiomyocytes, cTnT-∆K210 and -∆E160 cardiomyocytes showed distinct phenotypes in sarcomeric organization (Fig. 1C). A much higher percentage of heterozygous *TNNT2*^WT/∆K210^ cardiomyocytes showed DCM hallmarks, such as fewer myofibrils, more irregular disrupted sarcomere organization, and punctate cellular distribution of cTnT, which was even more pronounced in homozygous *TNNT2*^∆K210/∆K210^ cardiomyocytes (Fig. 1D). Ultra-structures analyzed by transmission electron microscopy (TEM) showed ∆K210 cardiomyocytes exhibited relative irregular Z-lines and expanded endoplasmic reticulum (Fig. 1E). In contrast, heterozygous *TNNT2*^WT/∆E160^ cardiomyocytes showed relative denser myofibrils with thickened Z lines. Homozygous *TNNT2*^∆E160/∆E160^ cardiomyocytes showed markedly disrupted myofibrils and Z-lines (Figs. 1E and S2).

**Figure 1:**
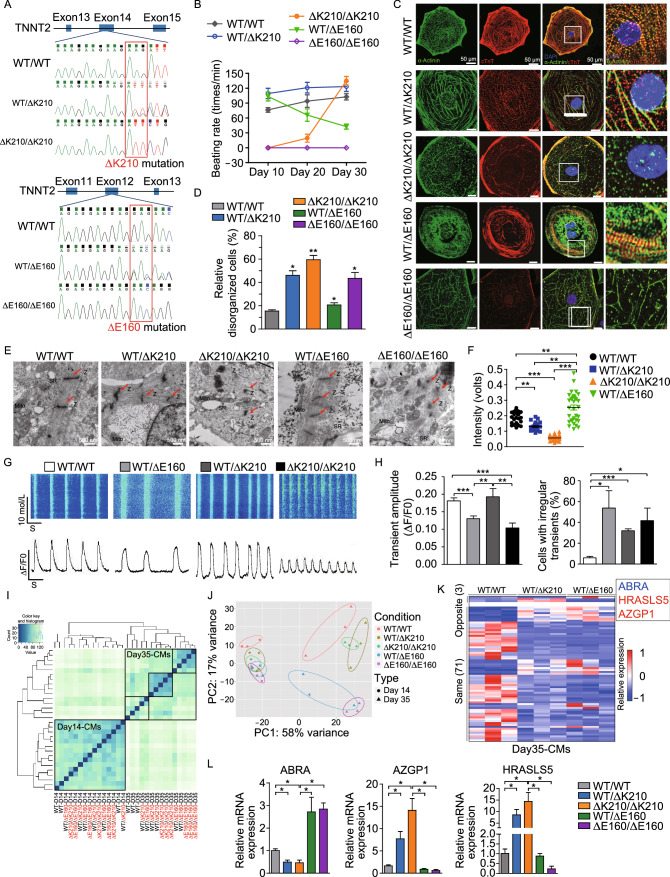
Generation of ∆K210 and ∆E160 mutant hESC lines, phenotypic characterizations of mutant cardiomyocytes, and identification of actin binding Rho activating protein (ABRA) as a candidate gene involved in the earliest disease divergence. (A) Sanger’s sequencing of PCR-amplified genomic DNA in wildtype (WT), heterozygous, and homozygous ∆K210 and ∆E160 hESCs confirmed deletion of AAG (K210) and GAG (E160) in exon 14 and exon 12, respectively, in the *TNNT2* gene. (B) The beating rate (times/min) of hESC-cardiomyocytes of different groups at day 10, 20, and 30 post differentiation. (C) Representative cellular myofilament organization of day 35 single WT, heterozygous and homozygous ∆K210 or ∆E160 cardiomyocytes immunostained with cTnT (red) and α-actinin (green). Scale bars, 50 μm. (D) The percentage of cells with disorganized sarcomeric pattern in each group at day 35 post cardiac differentiation. (E) Representative TEM images of myofibrillar organization of day 35 cardiomyocytes in each group. Z, Z-line. Red arrows indicate disorganized or thickened myofibrils. Scale bars, 500 nm. (F) Quantification of the relative spontaneous contraction forces for day 35 single hESC-cardiomyocytes in each group. (G and H) Representative Ca^2+^ line scan images of spontaneous Ca^2+^ transients and statistics of Ca^2+^ handling parameters of WT, ∆K210, and ∆E160 cardiomyocytes at day 35 post differentiation. (I and J) Whole transcriptomic RNA-seq profiles and principal component analysis (PCA) showed significant separation between WT, DCM ∆K210, and HCM ∆E160 cardiomyocytes at day 35 post differentiation. (K) Heatmap of the differentially expressed genes in day 35 WT, WT/∆K210 and WT/∆E160 hESC-cardiomyocytes. Compared with WT, genes showed opposite expression changes are listed in the box. (L) Quantitative PCR verification of *ABRA*, *AZGP1*, and *HRASLS5* expression in day 35 WT, WT/∆K210 and WT/∆E160 hESC-cardiomyocytes. *P < 0.05, **P < 0.01 and ***P < 0.001

For the cardiomyocyte function, both heterozygous and homozygous cTnT-∆K210 cardiomyocytes exhibited a reduced relative contraction force compared with WT cardiomyocytes, while cTnT-∆E160 cardiomyocytes showed increased contractility (Fig. 1F). Moreover, cTnT-∆K210 and -∆E160 cardiomyocytes exhibit a divergent Ca^2+^ handling properties and more irregular Ca^2+^ transients at day 35 post differentiation (Fig. 1G and 1H). Overall, these results showed that heterozygous ∆K210 and ∆E160 cardiomyocytes largely recapitulated cellular phenotypes of DCM and HCM respectively, while homozygous ∆K210 and ∆E160 cardiomyocytes showed more severe but abnormal cellular phenotypes.

Since DCM or HCM is progressively developed in patients’ lifetime, we hypothesized that genes involved in the earliest disease development and divergence will be valuable targets for interfering the disease. In this study, we used ∆E160 cardiomyocytes as a contrast to look for the key genes involved in the earliest disease progress during DCM development. Whole transcriptomic analyses of day 14 and day 35 ∆K210 and ∆E160 cardiomyocytes was performed using RNA-sequencing. Day 14 and day 35 cardiomyocytes exhibited distinct gene expression patterns and automatically grouped separately into two large clusters (Fig. 1I and 1J), suggesting they are at different maturation stages. Within the cluster of day 35 cardiomyocytes, it began to show differential gene expression patterns and almost automatically sub-clustered into WT, ∆K210 DCM, and ∆E160 HCM groups (Fig. 1I and 1J). To further narrow down the candidate genes in early disease development and divergence of DCM and HCM, we next compared those differentially expressed genes in day 35 ∆K210 and ∆E160 cardiomyocytes (the time that showed disease divergence) and searched for those genes exhibiting an opposite expression. As shown in Fig. 1K, there were 74 overlapped genes and among which only 3 genes showed opposite direction in expression changes. Of the 3 genes, ABRA (actin binding Rho activating protein, also known as striated muscle activator of Rho signaling (STARS)) were down-regulated in *TNNT2*^WT/∆K210^ DCM cardiomyocytes and up-regulated in *TNNT2*^WT/∆E160^ HCM cardiomyocytes. The other two genes HRASLS5 and AZGP1, which are strongly linked to metabolism, were up-regulated in *TNNT2*^WT/∆K210^ cardiomyocytes and down-regulated in *TNNT2*^WT/∆E160^ cardiomyocytes (Fig. 1K). Expression of *ABRA*, *AZGP1*, and *HRASLS5* in day 35 cardiomyocytes were further validated by quantitative PCR (Fig. 1L). Since ABRA was shown up-regulated in skeletal muscle hypertrophy and down-regulated in atrophy in human patients (Lamon et al., 2009), which is closely corresponding to heart muscle hypertrophy and dilation, we speculated that ABRA play an important role in the earliest disease divergence.

ABRA is a cardiac and skeletal muscle-specific actin-binding protein, which specifically localizes to the Z disc and M-line and directly binds actin (Arai et al., 2002). Previous studies demonstrated that ABRA activates the Rho signaling and serum response factor (SRF) mediated transcription by allowing nuclear translocation of MRTF-A and -B. ABRA also promotes actin polymerization by increasing the binding of G-actin to F-actin, strengthens myofilaments of striated muscle cells and modulates the heart response to stress signaling, which may function as a cytoskeletal intermediary of stress sensing and intracellular signaling regulation (Arai et al., 2002; Kuwahara et al., 2007; Wallace et al., 2012). Compared to WT cardiomyocytes, ABRA showed a striated localization pattern but tended to localize to the center of *TNNT2*^WT/∆K210^ cells and more disrupted in *TNNT2*^∆K210/∆K210^ cells (Fig. S3A). ABRA and cTnT protein levels were also decreased in cTnT-∆K210 cardiomyocytes (Fig. S3B and S3C). Overexpression of ABRA using lentivirus in cTnT-∆K210 cardiomyocytes also rescued the DCM-relevant cellular phenotypes, such as sarcomere disorganization, punctate distribution of cTnT and decreased contractile force (Fig. S3D–H). We reasoned that down-regulation of ABRA in cTnT-∆K210 cardiomyocytes reduced F-actin filament formation and may directly weaken myofilament structures, thereby leading to decreased cell contractility and DCM-relevant phenotypes.

We next investigated whether heart-specific expression of ABRA could reverse DCM phenotypes of the cTnT-∆K210 DCM mice *in vivo*. The results showed reduced ABRA protein in cTnT-∆K210 mice (Fig. S4A), staining the Z-lines with α-actinin and A-bands with tropmodulin (TMOD1) in cTnT-∆K210 mice further indicated the Z-lines were relative intact but A-bands were disrupted (Fig. S4B). Recombinant adeno-associated virus 9 (AAV9) with cardiac-specific cTnT promoter driving ABRA expression (AAV9-ABRA) was constructed and AAV9 with cTnT promoter driving luciferase (AAV9-Luci) served as a control. AAVs were injected at 2–3 days after birth through intraperitoneal injection. The luciferase protein was detected only in the hearts, which validated the virus performance (Fig. S5A).

Mice heart function was measured every month after AAV injection by echocardiography. It showed that impaired heart functions were effectively rescued in AAV9-ABRA treated cTnT-∆K210 mice at 3 months (Fig. 2A). Left ventricular ejection fraction (EF) and fractional shortening (FS) were reversed to a level close to WT values (Fig. 2B). The left ventricular end-systolic internal diameter (LVIDs) and LV posterior wall (LVPW) thickness of cTnT-ΔK210 mice treated with AAV9-ABRA were significantly improved (Figs. 2B and S5B). Heart size and heart weight/body weight value of AAV9-ABRA treated cTnT-ΔK210 mice were also much smaller and close to those of the WT (Fig. 2C and 2D). Histological analyses showed that AAV9-Luci treated cTnT-ΔK210 mice developed cardiac dilation with enlarged cardiomyocytes and increased myocardial fibrosis, while AAV9-ABRA treated mice reversed these typical DCM phenotypes and pathological cardiac remodeling (Fig. 2E). Moreover, AAV9-ABRA treatment restored the disrupted sarcomeric A-bands and M lines in cTnT-ΔK210 mice, although the sarcomere length still seemed compact (Fig. 2F). AAV9-ABRA treatment also improved overall survival rate of cTnT-ΔK210 DCM mice (Fig. 2G). These results indicated that the DCM phenotypes in cTnT-ΔK210 mice were markedly reversed by cardiac-specific ABRA expression. We next used the classical cTnT-R141W knock-in DCM mice (Juan et al., 2008) to examine whether ABRA heart-specific expression is effective in other cTnT mutation-induced familial DCM. As shown in Fig. S6, impaired heart functions in cTnT-R141W mice were rescued after AAV9-ABRA injection.

**Figure 2:**
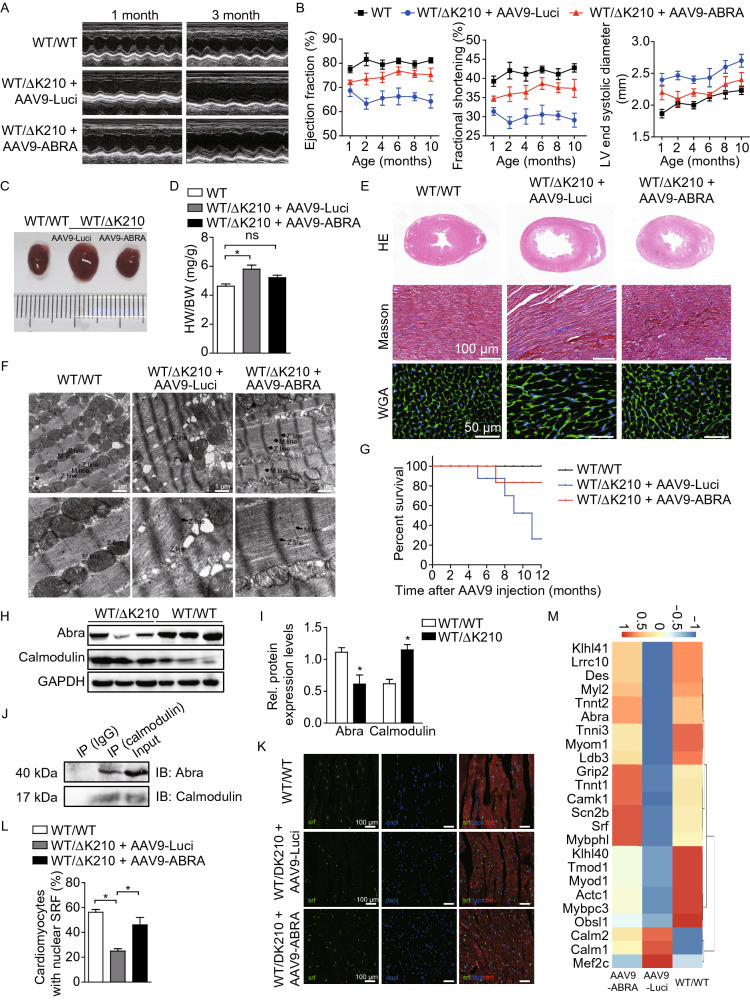
Cardiac-specific expression of ABRA rescued DCM phenotypes. (A) Representative M-mode echocardiography recordings of 1-month-old and 3-months-old WT, AAV9-Luci injected cTnT-∆K210, and AAV9-ABRA injected cTnT-∆K210 mice. (B) Serial echocardiographic measurements of ejection fraction (EF), fractional shortening (FS), left ventricular end systolic internal diameter (LVIDs) at different time points post virus injection. (C and D) Overall heart morphology and heart weight /body weight (HW/BW) ratio of the whole hearts 3 months after AAV9 virus injection. (E and F) Representative H&E, Masson’s trichrome, and WGA staining (E), was well as TEM images (F) of heart sections 3 months after AAV9 virus injection. Scale bars: 100 μm (Masson), 50 μm (WGA), 1 μm (TEM). (G) survival curves for WT, AAV9-Luci injected cTnT-∆K210, and AAV9-ABRA injected cTnT-∆K210 mice. (H and I) Western blot assessment and quantification of calmodulin and Abra protein levels in heart tissues of 3-months-old WT and cTnT-∆K210 mice. A pan-calmodulin antibody was used. (J) Co-immunoprecipitation of calmodulin and Abra from heart tissue extraction of WT mice. (K) Immunofluorescence staining of SRF in mice heart tissues of different groups 3 months after AAV9 virus injection. Scale bars, 100 μm. (L) Quantification of percentage of cardiomyocytes exhibiting positive nuclear SRF staining. (M) RNA-seq heatmap profiling of SRF-regulated muscle genes 3 months after AA9 virus injection. *P < 0.05. **P < 0.01.

In addition, our Ca^2+^ imaging results indicated that the cTnT-∆K210 mutation induced abnormal Ca^2+^ handling (Fig. 1G) and significantly upregulated calmodulin in the heart of cTnT-∆K210 mice (Fig. 2H and 2I). Previous study showed that the Ca^2+^-dependent protein calmodulin directly binds to the N-terminus of ABRA and negatively regulates muscle gene expression induced by the ABRA-serum response factor (SRF) pathway (Furuya et al., 2016), thereby linking Ca^2+^-signaling to ABRA-mediated gene expression. Immunoprecipitation assays showed that calmodulin binds to ABRA in cardiomyocytes (Fig. 2J). We then examined whether ABRA mediated SRF activity was changed in cTnT-∆K210 mice. Compared to WT mice, nucleus translocation of SRF was significantly reduced in heart tissues of cTnT-∆K210 mice (Fig. 2K and 2L). SRF-regulated muscle and contractile genes were significantly downregulated in cTnT-∆K210 mice and increased after AAV9-ABRA treatment as shown by whole transcriptome RNA-sequencing and further confirmed by QPCR (Figs. 2M and S7). These results suggest that the cTnT-ΔK210 mutation causes irregular Ca^2+^ handling in myofilaments and a resulted up-regulation of calmodulin, which negatively regulates ABRA-SRF activity in cardiomyocytes and compromised downstream muscle related gene expression.

Our current study showed that ABRA deficiency and compromised downstream SRF-regulated muscle gene expression play a role in cTnT mutation induced-familial DCM. ABRA could be a therapeutic gene for DCM patients carrying cTnT mutations. Our findings in this study may also serve as a new strategy in discovering early disease-associated genes for other mutation-caused familial DCM.

## FOOTNOTES

This work was supported by the National Natural Science Foundation of China (NSFC No.82070391, N.S.), the Postdoctoral Science Foundation (No.KLH1322109, B.L.), the Young Elite Scientist Sponsorship Program by CAST (2018QNRC001), the Haiju program of National Children’s Medical Center EK1125180102, and the National Key R&D Program of China 2018YFC2000202 (N.S.).

We thank Dr. Lianfeng Zhang at Peking Union Medical College for his kindly provision of the cTnT-R14W mice. We apologize to people whose work was relevant to but not cited in this study due to limited space.

Bin Li, Yongkun Zhan, Qianqian Liang, Chen Xu, Xinyan Zhou, Huanhuan Cai, Yufan Zheng, Yifan Guo, Lei Wang, Wenqing Qiu, Baiping Cui, Chao Lu, Ruizhe Qian, Ping Zhou, Haiyan Chen, Yun Liu, Sifeng Chen, Xiaobo Li, Ning Sun declare that there is no conflict of interests.

All institutional and national guidelines for the care and use of laboratory animals were followed.

The data, analytic methods, and study materials will be made available to other researchers for purposes of reproducing the results or replicating the procedure. The RNA-seq data have been deposited in GEO (Gene Expression Omnibus) with accession code GSE154096 and GSE154097.

B.L., Y.K.Z., and Q.Q.L performed and interpreted the experiments and wrote the manuscript. X.Y.Z. and H.H.C. performed H&E staining and MEA recording. C.X, Y.F.Z., Y.F.G, L.W., and W.Q.Q performed *in vitro* experiments. X.B.L., C.L., R.Z.Q and P.Z provided experimental advice. H.Y.C. performed echocardiography. Y.L. performed RNA sequencing and bioinformatics analyses. X.W. provided experimental advice. N.S. conceived the idea and experiments, provided experimental assistance, manuscript writing and funding support.

## Electronic supplementary material

Below is the link to the electronic supplementary material.

Supplementary material 1 (PDF 1470 kb)
