# Supplementary material for: Isogenic human pluripotent stem cell disease models reveal ABRA deficiency underlies cTnT mutation-induced familial dilated cardiomyopathy
Source: Protein Cell. 2021 Apr 22;13(1):65–71. doi: 10.1007/s13238-021-00843-w (PMC8776971; doi:10.1007/s13238-021-00843-w)
Supplement: Supplementary file 1 — Supplementary material 1 (PDF 1470 kb) [file 13238_2021_843_MOESM1_ESM.pdf]

Supplementary Materials

Isogenic human pluripotent stem cell disease models reveal ABRA deficiency underlies cTnT mutation-induced familial dilated cardiomyopathy

Materials and Methods

Animal care and use.

The cTnT-ΔK210 knock-in mice used in this study were constructed by VIEWSOLID Biotechnology Co. LTD (Beijing, China) using BALB/c background mice. The cTnT-R141W mice were kindly provided by Professor Lianfeng Zhang at Peking Union Medical College. All mice were housed in an animal facility with a 12-h light/dark cycle and free access to food and water. All procedures were performed in accordance with institutional guidelines and were approved by the Institutional Animal Care and Use Committee of Fudan University.

Culture and cardiac differentiation of normal and mutant hESCs

Normal and mutant H7 and H9 hESCs (Wicell Research Institute) were cultured on Matrigel (Growth Factor Reduced, BD Biosciences)-coated plates with mTesR-1 human pluripotent stem cell culture medium (STEMCELL Technologies) under conditions of 37℃, 95% air, and 5% CO2 in a humidified incubator. Cardiac differentiation of normal and mutant hESCs were performed according to previously described protocols (Lian et al., 2013). Briefly, hESCs were initially cultured in mTeSR1 medium on Matrigel-coated plates until they were ~80% confluent. On day

0-2, medium was supplemented with 6-10 μM CHIR-99021 (Selleck) in RPMI/B-27 without insulin. The medium was changed on day 3 to RPMI/B-27 without insulin for 24 h, followed by RPMI/B-27 without insulin supplemented with 5 μM IWR-1 (Sigma-Aldrich) for 2 days, then replaced with RPMI/B-27 without insulin for another 2 days. Cells were finally switched to RPMI/B-27 plus insulin medium. Beating cells were observed at day 7–8 after differentiation.

Construction of donor vectors for homologous recombination

A loxP-flanked PGK-puromycin cassette as well as 5'- and 3'- homology arms containing approximately 1600 bp of sequence homology flanking the TALEN-targeted site were inserted into the SYNPUC19CV backbone. The DNA fragment containing loxP-flanked PGK-puromycin cassette was polymerase chain reaction (PCR) amplificated from the pX261 plasmid (Addgene, 42337). ΔE160 and ΔK210 mutations were inserted into 5'-homology arm respectively using the Overlap Extension PCR. All the fragments were then assembled using Gibson Assembly® Master Mix (New England Biolabs) to generate the mutation containing targeting vectors. The introduction of the mutations and cassettes in donor vectors were confirmed by sequencing.

Contraction force measurements

Contraction force measurements were performed according to previously reported methods (Feaster et al., 2015). Briefly, single hESC-cardiomyocytes were cultured on a 0.4-0.8 mm thick mattress of undiluted Matrigel for 2-3 days, single beating cardiomyocyte with an elongated form were selected for the measurement, which

were visualized using a Zeiss CFM-500 inversion fluorescence microscope (Carl Zeiss). Contraction of single hESC-derived cardiomyocyte was detected by a video-based motion edge-detection system (Nikon) mounted on the microscope. Spontaneous contraction traces of cardiomyocytes were recorded with Video Sarcomere Length software (900B VSL, Aurora Scientific) coupled to the FelixGX software (Photon Technology International). Kinematic parameters were analyzed by FelixGX software and intensity of Y-axis reflected the relative contractility.

Immunofluorescence staining

Cells were fixed with 4% paraformaldehyde, permeabilized with 0.05% Triton X-100, blocked with 4% goat serum, and incubated with appropriate primary antibodies and Alexa Fluor conjugated secondary antibodies (Invitrogen). Cell nuclei were stained with 4, 6-diamidino-2-phenylindole (DAPI). Pluripotency of mutant hESCs were validated by immunostaining of Oct3/4 (Abcam), Sox2 (Abcam), SSEA-4 (Millipore), Nanog (Santa Cruz) antibodies. Alkaline phosphatase staining was performed using the Quantitative Alkaline Phosphatase ES Characterization Kit (Millipore) following the manufacturer's instruction. For characterization of differentiated hESC-cardiomyocytes, immunofluorescences were performed using cardiac troponin T (cTnT, Thermo Scientific), ACTN2 (Proteintech), myosin light chain (MLC-2A) (Proteintech), and myosin light chain (MLC-2V) (Proteintech) antibodies. Labeled cells were examined and imaged by a Leica confocal microscope (Leica). Cells with disorganized sarcomeric patterns were determined using the criteria described previously (27). Briefly, cells with punctuate cTnT pattern in greater than ¼ of the

total cellular area were evaluated as abnormal sarcomeric organization. For each image, this assess process was performed by two independent observers and averaged. Cell size was quantified using the ImageJ software package (National Institutes of Health) from mutant cells and side-by-side wildtype cells differentiated and cultured in the same condition. Cells were seeded at a lower density of ~20-30% for size calculation. At least 3 different batches of cells were calculated.

Calcium imaging

The contractile cardiomyocytes were incubated with Tyrodes Solution containing a Ca^{2+} indicator Cal- 520^{TM} and 0.02% Pluronic F-127 (AAT Bioquest) at 37 ℃ for 20 minutes, cells are washed with fresh medium before starting the experiment. Spontaneous Ca^{2+} transients were optically recorded with an LSM-710 laser scanning confocal microscope (Carl Zeiss) in the line (X-T)-scan mode. Ca^{2+} images were analyzed using the MATLAB software (MathWorks).

RNA-sequencing

Total mRNAs were isolated from normal and mutant H7-derived cardiomyocytes on day 14 and day 35 post differentiation using TRIZOL (Invitrogen) according to the directions. Poly(A) RNA was purified from total RNA using the MicroPoly(A)TM kit (Life Technologies). The cDNA library was prepared using an Ion Total RNA-Seq kit (Thermo Fisher Scientific). The mRNA-seq libraries were analyzed by Agilent Bioanalyzer and quantified using an Illumina Library Quantification Kit (KAPA Biosystems). A total of approximately 2500000 reads were obtained for each sample. Gene expressions were estimated as Fragment Per Kilobase of exon per Million reads

(FPKM) by cufflinks. We performed three biological replicates every line. The RNA-seq data have been deposited in GEO (Gene Expression Omnibus) with accession code GSE154096 and GSE154097.

Lentivirus production and gene overexpression in hESC-cardiomyocytes

ABRA cDNA was cloned into the target gene lentiviral plasmid. The HEK293T cells were plated in a 10 cm dish and transfected with target gene plasmid and packaging plasmids using Lipofectamine 2000 according to the manufacturer's protocol (Life Technologies). The transfected HEK293T cells were cultivated for 3 days and collected medium every 24 hours. To remove cell debris, collected medium was centrifuged at 6000 g for 15 min. The supernatant containing viruses was concentrated using ultrafiltration. Normal and mutant hESC-cardiomyocytes were seeded onto 6-well plates and infected with lentiviruses expressing ABRA gene. Lentiviruses expressing EGFP gene were used as the control. The transduction efficiency was confirmed 24 hours after infection using a fluorescence microscope.

Bioluminescence imaging

Luciferase activity in mice hearts was non-invasively assessed by in vivo bioluminescence imaging using luciferase assay reagents from Promega (Madison, USA). Anesthetized mice were intraperitoneal injected with D-luciferin and bioluminescence imaging were performed after 15 minutes using an IVIS Lumina K system (PerkinElmer, USA).

Recombinant AAV9 virus production and treatment

ABRA and Luciferase were separately cloned into ITR-containing AAV plasmid

harboring the cardiac specific cTnT promoter to obtain the pAAV.cTnT.ABRA and pAAV.cTnT.Luciferase plasmids, respectively. Adeno-Associated Virus serotype 9 (AAV9) was packaged in HEK293 cells with AAV9:Rep-Cap and pHelper, followed by an iodixanol gradient purification. AAV9 titer was determined by quantitative PCR. Constructs for ABRA and Luciferase were sent to Vigene Biotechnology (Shanghai) for AAV9 package and purification. Neonatal (2-3 days after birth) or adult (3 months old) mice were intraperitoneal injected with AAV9 viruses using a microliter from Hamilton. Mice received a total volume of 50 μl containing 1×10^{12} vg AAV9-Luci or AAV9-ABRA viruses in corresponding group. Cardiac function was examined using echocardiography after injection every month. Some mice were sacrificed and heart tissues subjected to histological, real-time RT-PCR, and immunoblot analyses 3 months after injection.

Echocardiography

Echocardiography was performed on unconscious mice lightly anesthetized by 1.5% isoflurane. Transthoracic echocardiography images were captured using a VisualSonics Vevo 2100 high frequency ultrasound system dedicated to small animal imaging (VisualSonics). M-mode recording was performed at the midventricular level. LV wall thickness, diastole and systole internal cavity diameters (LVIDd and LVIDs) were measured. Percent LV fractional shortening (%FS) and ejection fraction were calculated from M-mode measurements. The echocardiographer was blinded to mouse genotypes or treatments.

Histology analysis

For histological analysis, mouse heart tissues were fixed in 4% paraformaldehyde (PFA) for 24 h, dehydrated and embedded in paraffin for preparation of 4-um-thick histological sections. Rehydrated slides were stained with haematoxylin and eosin (H&E) to evaluate morphology and Masson's trichrome to evaluate cardiac fibrosis. For determination of cardiomyocyte size, deparaffinized and rehydrated heart sections were incubated overnight at 4 ℃ with Alexa Fluor™ 488 Conjugated-Wheat Germ Agglutinin and DAPI (Life Technologies, USA) to visualize myocyte membranes. Regions that included the circular shapes of capillaries were selected from the epicardial side of the LV-free walls. The mean size of cardiomyocytes was determined from 100 cells.

Transmission electronmicroscopy

The hESC-derived cardiomyocytes were immediately fixed in 2.5% glutaraldehyde solution for about 1 hour at room temperature, washed three times with PBS (15 min each) and post-fixed with 1% osmium tetroxide for 3 hours. After washed with PBS, samples were dehydrated in graded ethanol and then in propylene oxide for 10 min. Next, samples were embedded, sectioned at about 70 nm thickness, and stained with lead citrate for 8 min. Micrographs were captured using a PHILIPS CM-120 transmission electron microscope (PHILIPS). Scoring of myofibrils structure was performed by three independent observers and averaged. The following scores were used: 1: randomly scattered and fuzzy Z-structures, incomplete sarcomere, disrupted myofibrils; 2: distorted Z-line, not well organized sarcomere and myofibrils; 3: clearly visible and wide discrete Z-line, sarcomere with uniform length, well organized

myofibril alignment.

Statistical analysis

Two-tailed unpaired Student's t test was used to compare two normally distributed data sets. For comparison of >2 groups, 1- or 2-way ANOVA with either Tukey's or Bonferroni posttest was used. $P < 0.05$ was considered statistically significant. $^{*}P < 0.05$, $^{**}P < 0.01$ and $^{***}P < 0.001$. All measured data were presented as mean ± SEM unless indicated. Statistical analyses were performed with SigmaStat 3.5 (Systat Software), GraphPad Prism5.0 (GraphPad Software).

References:

Feaster, T.K., Cadar, A.G., Wang, L., Williams, C.H., Chun, Y.W., Hempel, J.E., Bloodworth, N., Merryman, W.D., Lim, C.C., Wu, J.C., et al. (2015). Matrigel Mattress: A Method for the Generation of Single Contracting Human-Induced Pluripotent Stem Cell-Derived Cardiomyocytes. Circ Res 117, 995-1000.

Lian, X., Zhang, J., Azarin, S.M., Zhu, K., Hazeltine, L.B., Bao, X., Hsiao, C., Kamp, T.J., and Palecek, S.P. (2013). Directed cardiomyocyte differentiation from human pluripotent stem cells by modulating Wnt/beta-catenin signaling under fully defined conditions. Nat Protoc 8, 162-175.

Extra Discussion

When ventricular dilation is already developed in patients, expressions of many genes will differ from those in normal hearts. However, most of them could be outcomes of cardiac remodeling and those earliest molecular events key for DCM development are prone to be obscured at this relative late disease stage. In this study, these *TNNT2* mutant cells we generated represent a very early stage both in embryonic and disease development. The diverged cellular phenotypes, Ca^{2+} handling, and contractility of the cTnT-ΔK210 DCM and cTnT-ΔE160 HCM cardiomyocytes

recapitulated disease features. Using HCM ΔE160 mutant cardiomyocytes as the contrast, we further discovered that the gene ABRA exhibited a diverged expression pattern in day35 ΔK210 and ΔE160 mutant cardiomyocytes, which may show an important role in the earliest disease divergence during DCM or HCM development. In this study, we found that both cTnT-ΔK210 hESC-cardiomyocytes and cTnT-ΔK210 mouse hearts exhibited ABRA deficiency, disorganized myofilaments, and decreased contractility. AAV9 mediated cardiac-specific expression of ABRA in cTnT-ΔK210 and cTnT-R141W mouse improved myofilament sarcomeric organization, reversed heart dysfunction, and especially, restored disrupted sarcomeric M-lines and A-bands. These results suggest that ABRA plays an important role in cTnT mutation induced familial DCM. Indeed, it is very interesting to see that expression of ABRA was significantly decreased in human idiopathic dilated cardiomyopathy patients (Figure S8).

Further, our data showed that SRF-regulated muscle proteins were all down-regulated in heart tissues of cTnT-ΔK210 mice. This may form a vicious cycle started from the cTnT-ΔK210 mutation, leading to abnormal Ca^{2+} handling of myofilaments, and increased expression of cytoplasmic calmodulin. Increased calmodulin inhibited ABRA and SRF signaling activities, subsequently led to down-regulation of ABRA protein expression, and gradually developed the DCM phenotype after repeated cardiac cycles of contraction-relaxation. Restoration of ABRA expression stopped this vicious cycle and significantly reversed SRF-regulated gene expression, therefore increasing the number and strength of myofilaments and

rescued the DCM phenotypes (Figure S9).

Supplementary Figures and Figure Legends

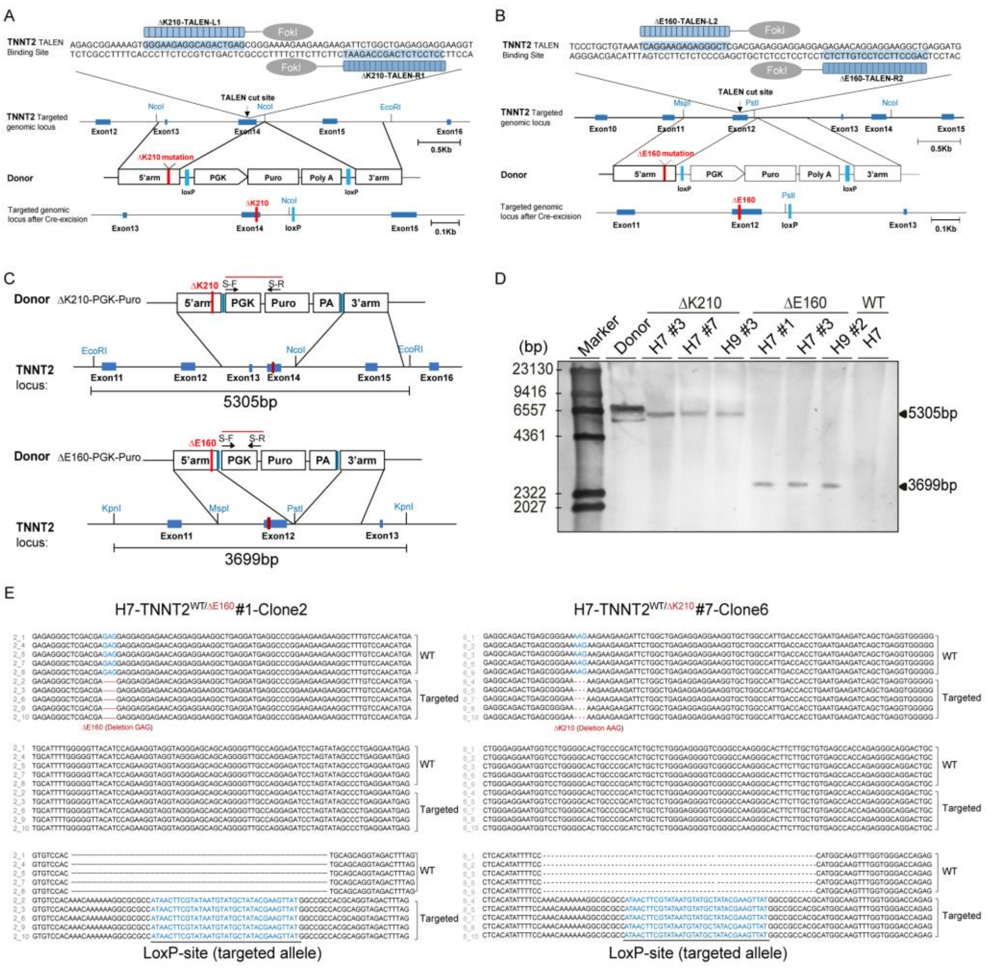

Figure S1. Design, validation, and assessing cutting efficiency for the TALEN pairs specifically targeting the *TNNT2* K210 and E160 locus. A and B, Schematics depicting the targeting strategies for introducing the ΔK210 (**A**) and ΔE160 (**B**) mutation into the human *TNNT2* gene by TALEN mediated homologous recombination. **C** and **D**, Southern blot analyses of hESC lines containing the correct ΔK210 or ΔE160 mutation after successful homologous recombination (before Cre-mediated excision of the selection cassette). The respective expected fragment size after KpnI or EcoRI digestion is indicated. **E,** Representative sequencing results

of TA cloning assays examining the genomic *TNNT2* E160 and K210 locus in puromycin selected hESC lines indicating a heterozygous pattern of the introduced mutations (*TNNT2*$^{WT/\Delta E160}$ and *TNNT2*$^{WT/\Delta K210}$).

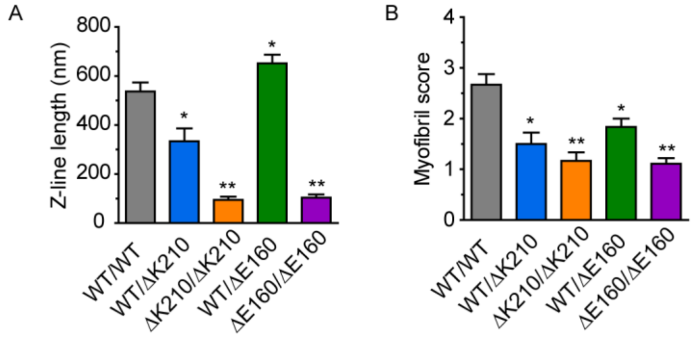

Figure S2. Quantification of the TEM images of myofibril organization in day35 cardiomyocytes. A, Z-line length of TEM cardiomyocytes sarcomere. **B,** Quantification of TEM myofibril structure scores.

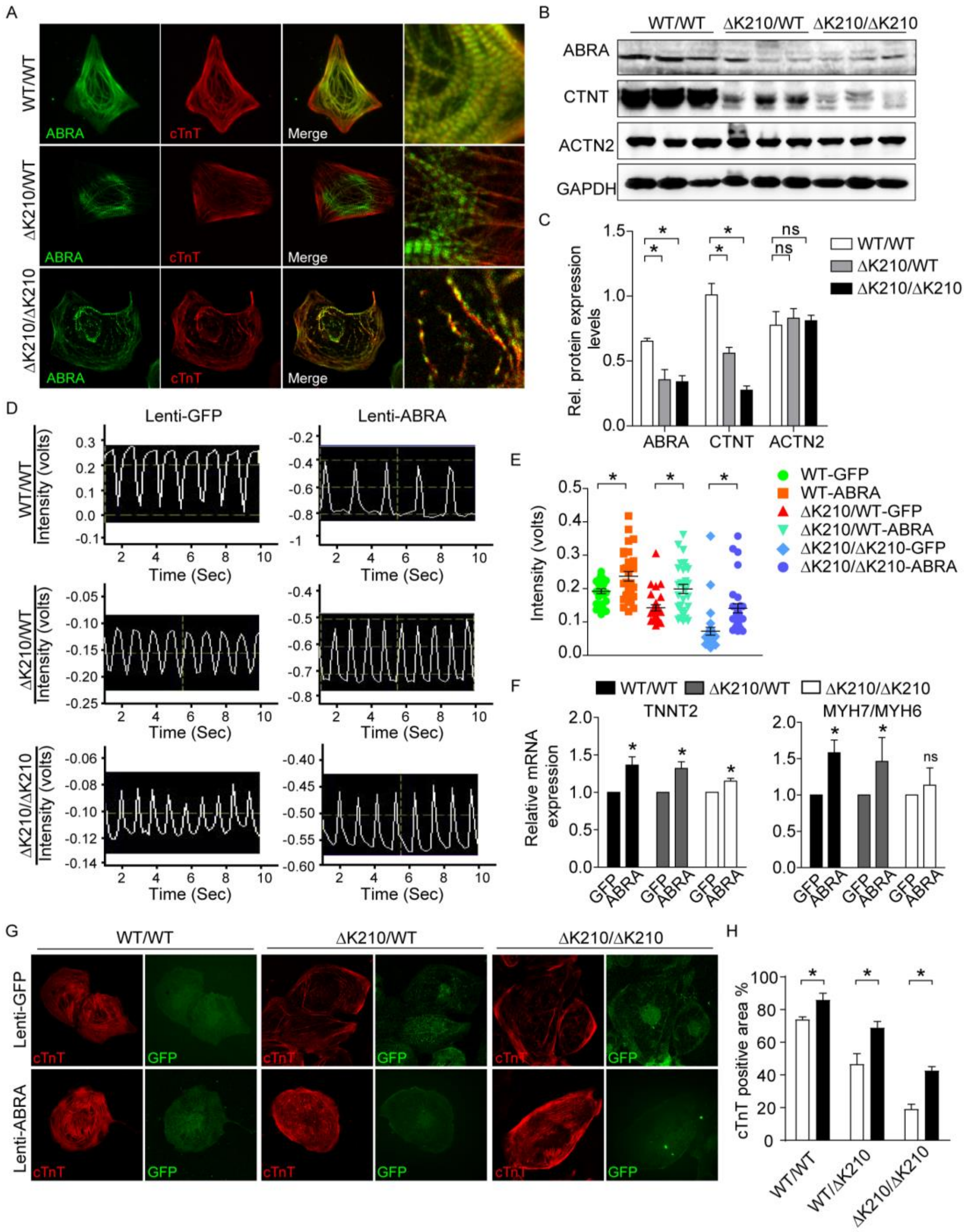

Figure S3. Overexpression of ABRA in WT and cTnT-ΔK210 hESC-derived cardiomyocytes. A, Double immunostaining of ABRA and cTnT (red) in cultured WT, WT/ΔK210, and ΔK210/ΔK210 hESC-cardiomyocytes. **B and C,** Detection and quantification of ABRA, cTnT, and ACTN2 protein expression at day35 post

differentiation by Western blotting. **D,** Representative video recording traces of spontaneous contraction of single WT, WT/ΔK210, and ΔK210/ΔK210 hESC-cardiomyocytes overexpressed with control EGFP or with ABRA. **E,** Statistics of video recording intensities showed increased contractile forces after ABRA overexpression in WT, WT/ΔK210, and ΔK210/ΔK210 hESC-cardiomyocytes. **F,** Relative expression of *TNNT2* and *MYH7/MYH6* after ABRA overexpression (control values were set to 1). **G,** Representative immunostaining of cTnT-positive myofilaments after EGFP (control) or ABRA overexpression. **H,** Percentage of cTnT positive area increased after ABRA overexpression. **P* < 0.05 and ***P* < 0.01.

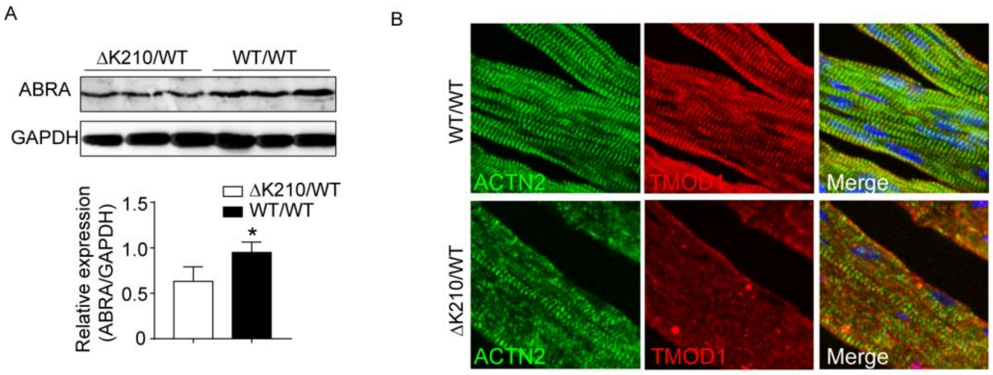

Figure S4. Heterozygous cTnT-ΔK210 mutant mice showed reduced ABRA protein expression and disrupted A-bands. A, Western blotting of ABRA protein expression level in new born WT and cTnT-ΔK210 mice. **B**, Immunostaining ACTN2 (green) and TMOD1 (red) in heart muscle of WT and cTnT-ΔK210 mice. Compared to the striated pattern in WT mice, Tmod1 staining were markedly irregular and scattered in heart muscle of cTnT-ΔK210 mice.

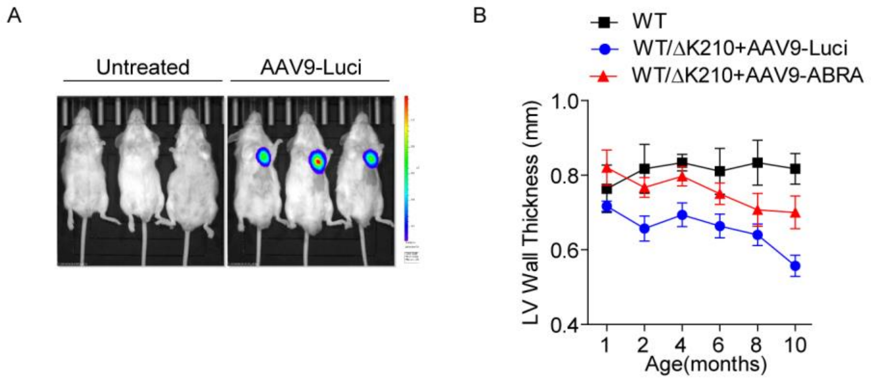

Figure S5. Cardiac-specific expression of ABRA in neonatal cTnT-ΔK210 mice. A, In vivo bioluminescence imaging of mice on day30 after AAV9-Luci injection indicated cardiac-specific expression directed by the cTnT promoter. **B,** Serial echocardiographic measurements LV wall thickness at different time points post virus injection.

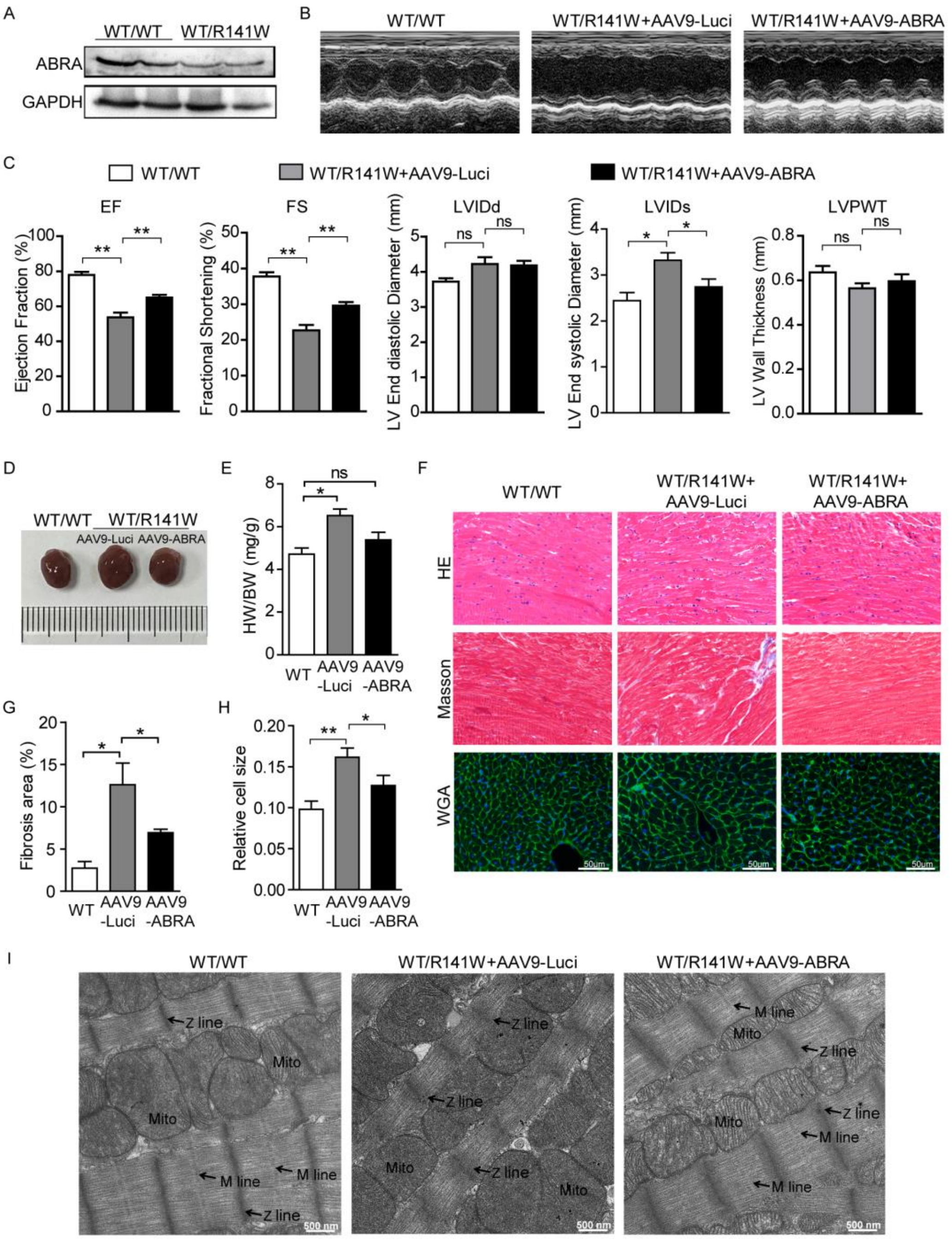

Figure S6. Heart-specific expression of ABRA reversed DCM phenotypes of the cTnT-R141W mice in vivo. A, Western blotting showed reduced Abra protein in cTnT-R141W mice compared with WT. GAPDH was used as the internal control. **B,** Representative M-mode echocardiography recordings of 3-months-old WT, AAV9-Luci injected cTnT-R141W (AAV9-Luci), and AAV-ABRA injected

cTnT-R141W (AAV9-ABRA) mice. **C,** Echocardiographic measurements of EF, FS, LVIDd, LVIDs, and LV wall thickness 3 months post virus injection. **D,** Overall morphology of the whole hearts from 3-months-old mice in each group. **E,** Heart weight and body weight (HW/BW) ratio of 3-months-old WT, AAV9-Luci injected cTnT-R141W, and AAV9-ABRA injected cTnT-R141W mice. **F,** Representative images of H&E (top), Masson's trichrome (center), and WGA immunofluorescence (bottom) staining of heart sections of WT, AAV9-Luci injected cTnT-R141W, and AAV9-ABRA injected cTnT-R141W mice. **G-H,** Quantification of interstitial fibrosis (**G**) and relative myocyte size (**H**) in each group. **I,** Representative TEM images of sarcomere ultrastructure in 3-months-old WT, AAV9-Luci, and AAV9-ABRA cTnT-R141W mice. Mito: mitochondria. Scale bars, 500 nm. *P<0.05. **P<0.01.

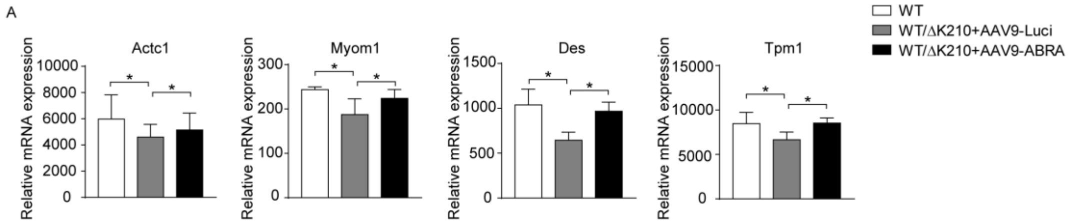

Figure S7. Quantitative-PCR validation of SRF-targeted muscle-related gene expression after heart-specific expression of ABRA. A, SRF-targeted muscle-related genes including *Actc1*, *Myom1*, *Des*, and *Tpm1* increased after ABRA overexpression. *P < 0.05.

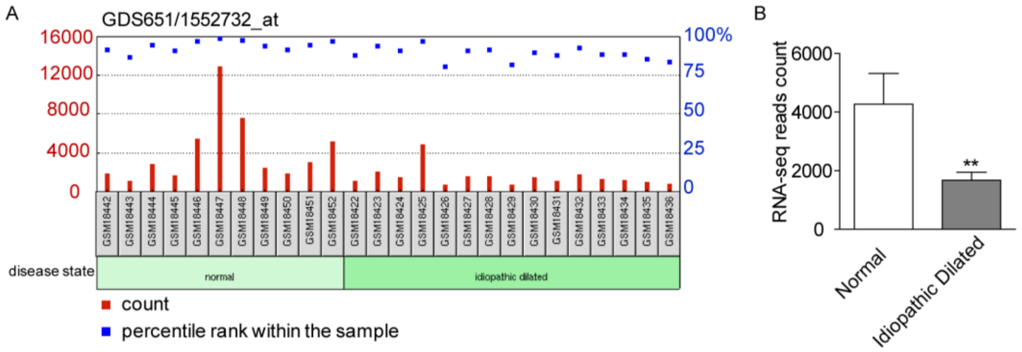

Figure S8, ABRA expression is down-regulated in human heart tissues of idiopathic dilated cardiomyopathy patients. A, Expression of ABRA in human heart tissues of normal individuals and of idiopathic dilated cardiomyopathy patients from GEO DataSets GDS651. **B,** The statistics of RNA-seq reads count value in A.

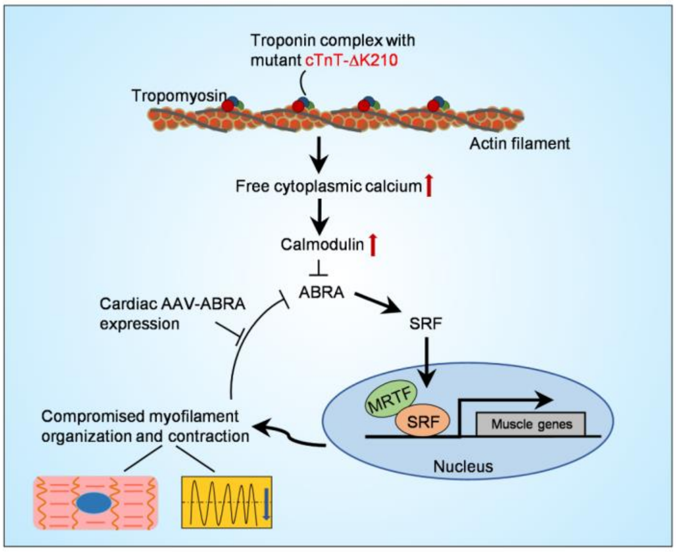

Figure S9. A diagram depicting the mechanisms underlying the function of ABRA in cTnT-ΔK210 mutation-induced familial DCM. cTnT-ΔK210 mutation resulted in abnormal Ca^{2+} handling of the myofilaments and increased expression of cytoplasmic calmodulin, which inhibited ABRA and SRF signaling activities, compromised sarcomeric organization and myocyte contractility. Restoration of

ABRA expression stopped this vicious cycle and rescued the DCM phenotypes.
